# Opioid Therapy Pharmacogenomics for Noncancer Pain: Efficacy, Adverse Events, and Costs

**DOI:** 10.1155/2013/943014

**Published:** 2013-09-18

**Authors:** Yan Xu, Ana Johnson

**Affiliations:** ^1^Queen's University, Kingston, ON, Canada K7L 3N6; ^2^Department of Population Health Sciences, Queen's University, Kingston, ON, Canada K7L 3N6; ^3^Institute for Clinical Evaluative Services, Queen's University, Kingston, ON, Canada K7L 3N6

## Abstract

Chronic non-cancer pain is a debilitating condition associated with high individual and societal costs. While opioid treatment for pain has been available for centuries, it is associated with high variability in outcome, and a considerable proportion of patients is unable to attain relief from symptoms while suffering adverse events and developing medication dependence. We performed a review of the efficacy of pharmacogenomic markers and their abilities to predict adverse events, dependence, and associated economic costs, focusing on two genes: OPRM1 and CYP2D6. Data sources were articles indexed by PubMed on or before August 6, 2013. Articles were first selected after review of their titles and abstracts, and full papers were read to confirm eligibility. Initially, fifty-two articles were identified. Of these, 17 were relevant to biological actions of pharmacogenomic markers and their effect on therapeutic efficacy, 16 to adverse events, 15 to opioid dependence, and eight to economic costs. In conclusion, increasing costs of opioid therapy have made the advances in pharmacogenomics an attractive solution to personalize care with unclear repercussions related to the impact on costs, morbidity, and outcomes. This intersection of pharmacoeconomics and pharmacogenomics presents a unique platform to further examine current advances in clinical medicine and their utility in cost-effective treatment of chronic pain.

## 1. Introduction

Chronic noncancer pain is a debilitating condition with high individual and societal costs [1–3]. Currently, the armamentarium of medications available to physicians in the treatment of chronic pain is restricted to nonsteroidal antiinflammatory drugs (NSAIDS), acetaminophen, adjuvants, and opioids [4], with few novel pharmacologic breakthroughs in the past 2 decades [5–7]. In recent years, population-based studies have demonstrated an increasing trend in prescription uptake of opioids among noncancer patients [8, 9]. A significant proportion of prescriptions to chronic pain patients consists of opioid medications [10]. During the time period encompassing 1997 and 2008, studies from the United States (U.S.) have shown that chronic opioid use in the general population ranges between 1.3% and 4.6% [11–13]. In a survey of pain management in 16 European countries conducted in 2003, Breivik and colleagues found that 28% of survey respondents used prescription opioids [10]. The countries reporting higher percentage of opioid use were no more satisfied with their medication pain control compared to those with lower prevalence of use [10]. In a study of Canadian patients on waitlist for multidisciplinary pain clinics spanning 2004 and 2007, over half of these patients were prescribed narcotic medications [14]. 

The predominant method of pain treatment continues to the Pain Ladder developed by the World Health Organization in the context of cancer care, which focuses on starting with nonopioids prior to the initiation of opioid therapy [15]. However, opioids are in the class of one of the most significant drivers of pharmaceutical costs in many jurisdictions. For example, sales of narcotic analgesics reached $12.3 billion in the U.S. and ranked 15th among therapeutic classes with the largest commercial market in 2011 [14]. In the same year, 238 million prescriptions of opioids worth $8.3 billion were filled in the U.S., the third most frequently prescribed class of medications in the country [16, 17]. Despite an enormous rise in spending and prescription, there is limited evidence on the efficacy of opioids in chronic noncancer pain management [18]. In a European survey on chronic pain, 15% of respondents felt that their medications were not very or not at all effective [10]. A systematic review by Chou and colleagues suggested limited efficacy of long-term opioid therapy over short-term treatment or placebo [18], while an evidence review by the Institute of Medicine concluded that the effectiveness of opioids as pain relievers, especially over the long term, is somewhat unclear [3].

Several factors have been put forth to explain the rise in opioid prescriptions for chronic noncancer pain that began in the 1980s and became dominant one decade later [19]. First, reservations against alternative pain therapies were growing, especially those related to adverse events associated with long-term use of nonsteroidal antiinflammatory drugs [20]. Second, aggressive and, at times, misleading product marketing by the manufacturers further contributed to this upsurge [19]. Third, there has been a widespread belief that opioid therapy carried a low risk of addiction potential, despite the inadequate quality of evidence supporting these claims [20].

Over the past two decades, the increasing knowledge of our genetic variation has facilitated the emergence of a new field known as pharmacogenomics [21]. The study of specific genetic variations that influence metabolism, response and action, and pharmacogenomics has the potential to change current and future medical practices [22–24]. Evidence suggests that the intersection between pharmacoeconomics and pharmacogenomics presents a unique platform to examine current advances in clinical medicine and their utility in cost-effective treatment of chronic pain. Questions still remain on the efficacy of pharmacoeconomic markers, adverse events, and dependence, as well as economic costs.

In this paper, we review the literature and examine the following two questions in particular.

1. What are the current pharmacogenomic markers that predict opioid response and susceptibility to adverse, focusing in particular on two genes in opioid therapy that have received most investigations to date: OPRM1 and CYP2D6?
2. What are the general healthcare costs related to ineffective opioid therapy, adverse events, and medication dependence?

## 2. Materials and Methods

We searched PubMed on August 6, 2013 to include all articles indexed to date. Search terms included disease keywords (“opioid”, “adverse events”, “dependence”, “therapeutic efficacy”), biological terminology (“polymorphism”, “allele”, “genetic determinant”), and health economics terms (“economics”, “pharmacoeconomics”, “resource utilization”, “costs”) to capture the literature on clinical outcomes and financial burden. Search results were limited to publications on human data in the English language. Article titles and abstracts were assessed to identify publications with specific relevance to opioid pharmacogenomics, as well as measurement of disease burden, direct medical costs, treatment cost of adverse events, and dependence associated with opioid therapy in noncancer pain. The electronic search was supplemented by manual searching of the reference lists in each relevant article to identify other papers that may have been missed in the review. Articles were first selected after review of their titles and abstracts, and the full paper was read to confirm eligibility for inclusion.

## 3. Results and Discussion

Fifty-six articles were identified in the initial review using the search strategy delineated above. Of these, pertaining specifically to OPRM1 and CYP2D6, five were relevant to biological actions of pharmacogenomic markers and their population prevalence, 14 on their effect on therapeutic efficacy, and 22 to adverse events and dependence. Seven articles specifically addressed economic costs of opioid therapy in general, while seven were relevant to frequency and impact of adverse events. The manual search yielded one relevant textbook reference that met the inclusion criteria.

First, we present the features of pharmacogenomics markers associated with opioid therapy as follows: mechanism of action and efficacy, followed by adverse events and dependence associated specifically to OPRM1 and CYP2D6. We then present a summary of the studies relating to the economic costs of opioid therapy in general.

### 3.1. Pharmacogenomic Markers Associated with Opioid Therapy

#### 3.1.1. OPRM1 Receptor

*Mechanism of Action and Efficacy.* Since its original description in 1998, single nucleotide polymorphisms (SNPs) in the human *μ*-opioid receptor (OPRM1) have been investigated for their role in human nociception, opiate efficacy, and addiction [25–27]. The variant most intensely studied is 118 A > G, with G allele prevalence of 7.4% to 15.3% in the Caucasian population, 1.6% in the African American population, and up to 48.6% in the Asian population [28–30]. In recent years, association between OPRM1 allele and therapeutic efficacy of opioids has been reported in cancer patients and postsurgical settings, where the 118G allele has been proposed to result in lower analgesic effect of opioids and higher morphine requirement [31–35]. In a study of 99 cancer patients with adequate pain control, Klepstad and colleagues compared morphine doses and OPRM1 genotypes and found a 2.3-fold difference between morphine doses of homozygous 118G patients compared to the 118A wild-type group, despite comparable baseline parameters in pain, cancer quality of life scores, and minimental examination scores [31]. In postsurgical studies, Chou and colleagues demonstrated that among patients undergoing total knee replacement and abdominal hysterectomy, those who are homozygous for 118G were more likely to have higher morphine consumption and demand, though the differences were less pronounced than those seen in malignant pain [32, 33]. Using two validated pain-induction methods, Oertel and colleagues demonstrated SNP as a determinant of pain control in alfentanil, in which 118G carriers required 2.2 times higher opioid to reach 50% increase in analgesia [36]. More relevant to noncancer chronic pain, Lotsch and colleagues demonstrated a trend towards higher pain associated with 118G allele in a group of 352 patients at outpatient pain centres [37]. In addition to its effects on morphine and alfentanil, suggestions have been made that central nervous system effects of methadone are also modulated by these SNPs [38].

*Adverse Events and Dependence.* In opioid-related adverse events, 118G SNP in the *μ*-opioid receptor has been proposed to have a protective role against opioid-induced vomiting and nausea, as well as central nervous system (CNS) depression [36, 39, 40]. Adverse event rates have been often reported as an adjunct rather than a primary end-point and have been sparsely published. One study found an 8.4% reduction in incidence of nausea among 588 women receiving morphine after caesarean [40], whereas lower nausea and sedation scores among A118G heterozygous in comparison to homozygous 118A patients were reported in the postsurgical setting, [39]. Oertel and colleagues, evaluating CNS effects, reported among healthy participants, that respiratory depression was significantly reduced among homozygous 118G carriers, as opposed to heterozygotes or 118A homozygotes [36]. The observations of polymorphism-dependent drug effect hold biological appeal, as *μ*-opioid receptor is the primary binding site of opioid drugs, with evidence from in-vitro studies demonstrating lower OPRM1 expression in 118G carriers [41]. However, reports indicating null or contradictory effects of these therapeutic observations have also emerged [42, 43]. In the 2009 metaanalysis of 9 studies with over 1900 combined patients, Walter and Lotsch concluded that no strong data exist for the 118G allele to predict opioid dosing and that the current state of evidence does not warrant personalizing decisions of pain therapy on this SNP of the OMRP1 gene [44].

The involvement of *μ*-opioid receptor SNPs in substance dependence has been suggested since its characterization [26]. While the receptor polymorphism has garnered much attention and research in the realm of its relationship to alcohol dependence [26, 45–47], perhaps in part due to the low incidence of iatrogenic opioid abuse [48], the investigation of pharmacogenomics in opioid dependence is an important one, especially for individuals with high preexisting risk for developing dependence prior to initiation of therapy, as well as the high costs of opioid abuse and diversion. A variety of *μ*-opioid receptor SNPs have been associated with opioid addiction, including C17T, A118G, and C691G [49–51], though these relationships are not consistent [29, 52]. Recently, attention has focused on spliced variants of *μ*-opioid receptors, specifically the 6TM isoform, in modulating opioid dependence [53]. It remains to see whether associations of spliced variants with substance dependence will be maintained with further clinical studies.

#### 3.1.2. CYP2D6

*Mechanism of Action and Efficacy.* The cytochrome P450 (CYP) superfamily is the major enzyme involved in phase I biotransformation of chemicals in the body, with both activating and inactivating properties. Of the diverse repertoire of CYPs that contains 57 genes, three groups (CYP2D6, CYP3A, and CYP2C) are notably involved in opioid metabolism; in particular, CYP2D6 is mainly involved in the biotransformation of codeine, hydrocodone, oxycodone, and tramadol [54]. In codeine metabolism, roughly 10% is converted via CYP2D6 to morphine, the key product involved in analgesic effect of the drug [55]. There is high genetic variation within the CYP2D6 gene, which has led to several medications being withdrawn from the market [56]. In the context of opioid metabolism, the presence of an ultrarapid metabolizer (UM) genotype is associated with high morphine levels, and the opposite is observed in the poor metabolizer (PM) genotype [55]. The prevalence of UM genotypes ranges between 0.9% and 6.9% in the general population; despite their low prevalence, carriers are characterized by an overwhelming amplification of CYP2D6 metabolizing capacity [57]. Prevalence of PM genotypes is somewhat similar, seen in 5–13.5% of Caucasians and up to 1% of Asians [58], though some reports have suggested higher frequencies [59]. Among PM carriers, opioids such as codeine and tramadol lack analgesic efficacy, and patients may be suspected of drug noncompliance and dependence when they report nonalleviation of pain [60–64]. In a randomized, double-blind study of 48 children given codeine after adenotonsillectomy, a significant relationship between plasma morphine levels and metabolizing genotypes was reported, whereby PM phenotypes were associated with lower serum morphine levels [62]. Further, a study of 300 postabdominal surgery patients found that patients with PM genotypes required 33% higher secondary loading dose of tramadol in recovery room when compared to UM patients [63]. Finally, in an evaluation of hydrocodone efficacy using experimental pain assessments in a small group of healthy volunteers, a 5- to 20-fold reduction in pain tolerance was found among PM carriers when compared to UM carriers [64].

*Adverse Events and Dependence.* UM carriers are at risk of developing toxic effects from excessive metabolite levels when given a therapeutic dose of opioid [65]. The dangers of this pharmacogenomic phenomenon were demonstrated in recent case reports of life-threatening events that occurred as result of codeine use in UM individuals [66, 67]. Though initially described in codeine, toxicity sequelae of CYP2D6 UM genotype extend to other opioids biotransformed using this enzymatic pathway, as it was described in a case of tramadol-related respiratory depression [68]. Further, opioid UM genotype can exert significant effects beyond the gene carriers themselves. Koren and colleagues described a case of neonatal morphine exposure and death following breastfeeding by a mother with UM phenotype prescribed Tylenol-3 for postpartum analgesia [69]. The case received widespread media coverage [56, 70] and has since prompted regulatory warnings on the use of codeine in breastfeeding mothers [71, 72]. Individuals who are PM in CYP2D6 are known to be protected against potential opioid dependence [73], while individuals with UM in the enzyme may be at a high risk [58]. 

In 2011, a retrospective database study reported on the 6-month healthcare utilization and costs associated with drug-drug interactions involving opioids metabolized via the CYP450 pathway, evaluating primary care physician, outpatient, and emergency room visits, as well as inpatient lengths of stay. Compared to individuals who were not coprescribed with an additional CYP450-metabolized drug, low-back pain patients using an interacting medication had significantly greater ambulatory visits and accrued over $700 in excess medical costs per patient [74]. A similar analysis in osteoarthritis patients revealed cost differences greater than $1,000 per patient [75]. While the change in therapeutic opioid levels due to the use of competing CYP450 drugs is an iatrogenic effect, similar mechanism is at the root of natural polymorphisms that give rise to the varying metabolic speed of codeine and other opioids that utilize this pathway. 

### 3.2. Economic Costs of Opioid Therapy

All studies found in this literature search pointed to an increase in spending on opioids over time. For example, U.S. sales of opioids increased by 127% between 1997 and 2006, with oxycodone and hydrocodone leading in the surge at 732% and 244%, respectively [8]. From 1998 to 2003, spending on opioids among medicaid enrollees expanded by 300% to reach $1.2 billion (year of costing, 2003), roughly 4% of the program's total spending on prescription medications [1]. Beyond prescription costs, healthcare expenditure on opioid users is high, with an annual cost of $23,049 per enrollee that includes ambulatory and emergency room visits, inpatient admissions, pharmacy costs, and investigations, more than four-fold higher than matched nonopioid users [13] This is attributed to the greater contact with the healthcare system experienced by chronic opioid users, whose utilization of ambulatory visits, emergency room visits, and inpatient lengths of stay is 2.8-, 2.5-, and 4.0-times greater than that of the users who do not take opioids [13]. In a Canadian study of pain clinic waitlists, mean cost for each patient was $3112 per month [2]. In Ontario, Canada, prescription of opioid analgesics increased by 29% between 1991 and 2007 [76]. 

Studies presented significant costs associated with adverse events. Common adverse events in opioid therapy include nausea, vomiting, and constipation, as well as central nervous system manifestations such as dizziness, confusion, and sleep disturbance [77]. These directly interfere with patient adherence and are attributed to opioid discontinuation in approximately 25% of patients [48, 78]. Kalso and colleagues demonstrated an upper limit of pain improvement on opioids at 30%–35% [78]. In surgical hospitalizations, opioid-related adverse events contribute to 7.4% and 10.3% increase in cost and lengths of stay, respectively [79]. Furthermore, a Cochrane systematic review found that 10.3% of patients prescribed with opioids for chronic noncancer pain discontinued their therapy due to the inefficient pain relief [48]. Of note, healthcare costs associated with adverse events have been found to represent 12%–61% of baseline opioid prescription costs, a significant burden considering the magnitude of narcotic analgesic spending [80]. From the healthcare system costs, comparisons of oxycodone, morphine, and fentanyl reported annual treatment costs for adverse events as to be ranging from US $303.19 and US $331.79 per patient, while a similar study investigating inexpensive antiemetics reported the cost of treatment to range between U.S. $85.38 and $141.65 per year [77]. Frei and colleagues, taking into account the duration of treatment for each patient and the prevalence of adverse events, reported adverse event costs of 575 to 1150 Danish kroners per patient in a year or U.S. $123.85–$247.70 [80]. Finally, the literature has shown that opioid overdose is the second leading cause of unintentional death in the U.S. [81], while the estimated total healthcare costs attributed to prescription opioid abuse are estimated at US $25.0 billion [82]. 

In sum, the staggering health care economic costs related to opioid use, adverse events, and abuse point to an urgent need for strategies that prospectively identify, appropriate treatment choice and dosing, while reducing negative drug reactions. 

## 4. Implications and Future Directions

In this paper, we found diverse levels of evidence for two putative markers of opioid efficacy, adverse events and dependence, against the backdrop of enormous human and healthcare resource costs associated with opioid use and abuse. In particular, two candidate genes receiving most investigations in opioid pharmacogenomics, OPRM1 and CYP2D6, were reviewed for evidence-base associated with their utility in genetic screening for tailoring of opioid dosing, as well as the occurrence of adverse events and therapy dependence. Despite the numerous descriptions of positive association between OPRM1 118G allele and increased opioid requirement, emergent contradictory reports have rendered this relationship currently inconclusive. Meanwhile, the relationships between CYP2D6 metabolic genotypes, opioid usage, and adverse toxicity have been well established and may warrant consideration in selecting populations with high frequency of CYP2D6 variants. The relationship between this genotype and opioid adverse events is further evidenced by the recent FDA decision to mandate label changes to restrict use of codeine in pediatric posttonsillectomies and adenoidectomies [83]. The literature showed that over the past decades, the use of opioids has dramatically increased as a method of treatment for chronic noncancer pain, which is associated with a significant increase in healthcare resources. The evidence discussed in this paper demonstrates that, currently, results from both clinical trials and bedside practices have overwhelmingly pointed to both the diversity of pain treatment outcomes and the significant proportion of patients who are unable to attain relief from their symptoms [65].

The enormous cost associated with opioid-related effects prompts dialogue on the feasibility of using pharmacogenomics as a tool to improve effectiveness and reduce adverse outcomes. Genotyping of candidate loci in the context of preventing opioid adverse event or therapeutic failure holds conceptual appeal. Of paramount interest to consumers, clinicians and policymakers, however, is whether such screening will improve patient outcomes in the “real-world” and whether these practices will be cost-effective. While the Clinical Pharmacogenetics Implementation Consortium has put forth genotype-based dosing guidelines on tramadol, oxycodone, and codeine [84], none has evaluated the effectiveness of a genotyping service on mortality and length of stay, or the other quality of care and healthcare resource endpoints. The available literature on warfarin suggests that selective genotyping in patients positively influences outcomes and can be cost-effective [24, 85]. Of note, while the recent FDA decision on codeine prescription in postadenotonsillectomies did not recommend use of routine genotyping for practicality reasons [83], the cost-efficacy balance may look favorable with introduction of newly reported array technologies capable of providing genetic polymorphism results for approximately 16*¢* per genotype, excluding implementation and human resource costs [86]. With decreasing costs of DNA sequencing, the genetic screening costs in many diseases have become reasonable: the cost of analyzing 3 BRCA gene mutations commonly found in Ashkenazi Jews, a high-risk population for the development of breast cancer, is $385 [87]. 

When discussing findings of the current review, some potential limitations ought to be acknowledged. Available studies examining pharmacoeconomics of adverse event treatment costs vary widely between countries, treatment medications used, and cost estimation models, which may restrict the comparability of findings. As well, economic cost evaluations of opioid therapy failure are likely an underestimate, as most economic evaluations of opioids may not capture costly but potentially less recognized adverse events, such as bone fractures secondary to opioid-induced nausea and confusion [88], opioid-induced osteoporosis [89], or life-threatening events associated with ultrarapid opioid metabolism and subsequent toxicity [90]. 

In conclusion, there is paucity of pharmacoeconomic studies specifically addressing efficacy, adverse events, and dependence associated with genetic markers of opioid therapy. With the rise in personalized medicine, the widespread use of genomic association in clinical practice remains to be seen [58, 65, 91–93]. The concept that individuals with divergent genetic polymorphisms respond differently to therapeutic compounds has gained widespread attention. As of 2011, 102 drugs that received FDA approval contain pharmacogenomic information on their labels [92, 94]. There is a dearth of pharmacogenomics data to predict dosing and outcomes, although a pharmacogenetic approach to warfarin dosing, for example, has been shown to predict initial dose and short duration in achieving stable therapeutic effects [85]. However, each and every genome of each patient receiving opioids would need to be sequenced, and consequences of rare mutations would need to be evaluated with respect to drug response. As further data become available, there is a promise to examine the impact of genome marker through modeling. This could suggest a promising new way to improve the benefit-harm profile and the net health benefit of medications [95, 96]. 

Polymorphisms that predict therapeutic benefit or risks are often rare in the general population, and their prevalence appear to vary between studies. Further, for candidate genes such as OPRM1, clinical implications of their polymorphisms are not yet conclusive. There is evidence for the role of CYP2D6 in codeine-associated toxicities [83], and future studies ought to incorporate economic evaluations to determine under what circumstances such screening strategies are cost-effective. Assessing the practicality of adopting such genetic tests is especially relevant in the context of determining a patient's opioid addiction risk, in combination with tools recommended by the Canadian guideline for Opioid Use for Pain [97]. 

While drug policy makers, clinicians, and patients await confirmation of the clinical and financial feasibility of pharmacogenetic testing for opioids, this study has contributed towards filling the gap in the pain medicine literature on the economic landscape of such tests. Identification and evaluation of genetic markers that provide prognostic information, matched with the advent and dissemination of new pharmacogenomic technologies, could hold promise of cost-effective tools that are capable of assisting clinicians in optimizing care and reducing unnecessary costs in opioid therapy for noncancer pain if used judiciously. On the other hand, the half hazard introduction of diagnostic measures into clinical practice could increase costs with uncertain outcomes.
